# Diagnosis Using CCTA and Management of Anomalous Right Coronary Artery from the Opposite Sinus

**DOI:** 10.1155/2016/7685360

**Published:** 2016-07-11

**Authors:** Asma Mursleen, Gregory Hartlage, Aarti Patel, Eric E. Harrison, C. Alberto Morales

**Affiliations:** Memorial Hospital of Tampa, Tampa General Hospital, University of South Florida, Cardiology Clinic, 602 Audubon, Suite B, Tampa, FL 33609, USA

## Abstract

Coronary anomalies can be observed in 1–1.2% of all angiograms performed. Majority of coronary anomalies are benign and do not lead to cardiac ischemia; however anomalous coronary arteries from the opposite sinus (ACAOS) are often associated with sudden cardiac deaths, typically in 0.11–0.35% of individuals who participate in vigorous physical activity (Peñalver et al., 2012). Left and right ACAOS have an incidence of 0.15% and 0.92%, respectively. Left ACAOS are often associated with higher incidence of sudden cardiac death; this could be secondary to greater territory of myocardial perfusion by the left coronary artery. ACAOS are often asymptomatic and initially present as sudden death following exertion in young athletes. The management of left ACAOS is clear and surgery is usually indicated. However there is a lack of consensus on the management of certain cases of right ACAOS. In this paper a case of 20 yo M with right coronary artery from left sinus is going to be presented with a discussion on pathophysiology, diagnosis, and management.

## 1. Case

**Figure 1:**
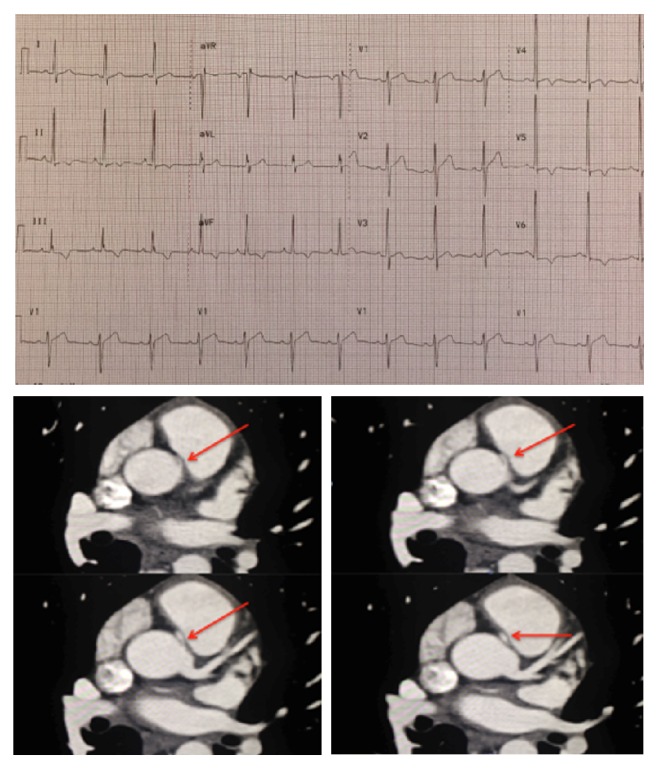
EKG showing probable left ventricular hypertrophy and nonspecific ST/T wave changes in the inferior and anterior lateral leads. CCTA showing the right coronary artery originating from the left coronary sinus.

A 20-year-old Caucasian male presented to the cardiac clinic for evaluation of chest pain and elevated blood pressure. His chest pains were sharp, occurred randomly, and were mostly nonexertional. He also had an abnormal EKG demonstrating nonspecific ST/T wave changes (see Figure 1). His past medical history was unremarkable. He is a smoker and drinks alcohol socially and denied illicit drug usage. An echocardiogram performed in the office was normal, with the coronary arteries arising from the appropriate cups.

A cardiac computed tomography angiography (CCTA) was performed revealing anomalous origin of the right artery from the left coronary cusp. The proximal portion had an acute-angle take-off with an intramural course with a 70–80% stenosis. The vessel also coursed between the aorta and pulmonary arteries (see Figure 1).

## 2. Discussion

Anomalous coronary arteries from the opposite sinus (ACAOS) are usually asymptomatic; however they can initially present as syncope or even sudden cardiac death in up to 0.35% of the cases [1]. It is important to include ACAOS in the differential diagnosis of a young patient complaining of any cardiopulmonary symptoms as early diagnoses and management of ACAOS can likely prevent sudden cardiac death. Management of left ACAOS is clear and surgery is usually recommended. Right ACAOS with clear evidence of cardiac ischemia can also lead to sudden death and surgery is suggested. In this case the patient had a right ACAOS with atypical symptoms; the management of which is controversial.

Right ACAOS can be further classified into interarterial, subpulmonic, and retroaortic course. Interarterial ACAOS originates above the pulmonary valve and courses between the aorta and pulmonary arteries and is associated with a higher prevalence of chest pain and cardiac events [2]. Exertion can lead to engorgement of vessels and possible mechanical compression of the anomalous coronary artery leading to decreased perfusion and increased risk of sudden death [2]. An acute-angle take-off is associated with kinking of the coronary artery at its origin, affecting blood flow as well.

An intramural course is associated with possibly the highest risk of adverse cardiac events. The proximal intramural segment intussuscepts into the aortic wall leading to coronary artery hypoplasia of the involved segment within the aortic wall. Systolic compression of the aortic wall laterally compresses the intramural stenotic coronary segment [3]. The hypoplastic intramural coronary segment is usually ovoid and lateral compression of an ovoid artery leads to a smaller area in comparison to the same lateral compression of a circular artery of the same circumference [3].

**Figure 2:**
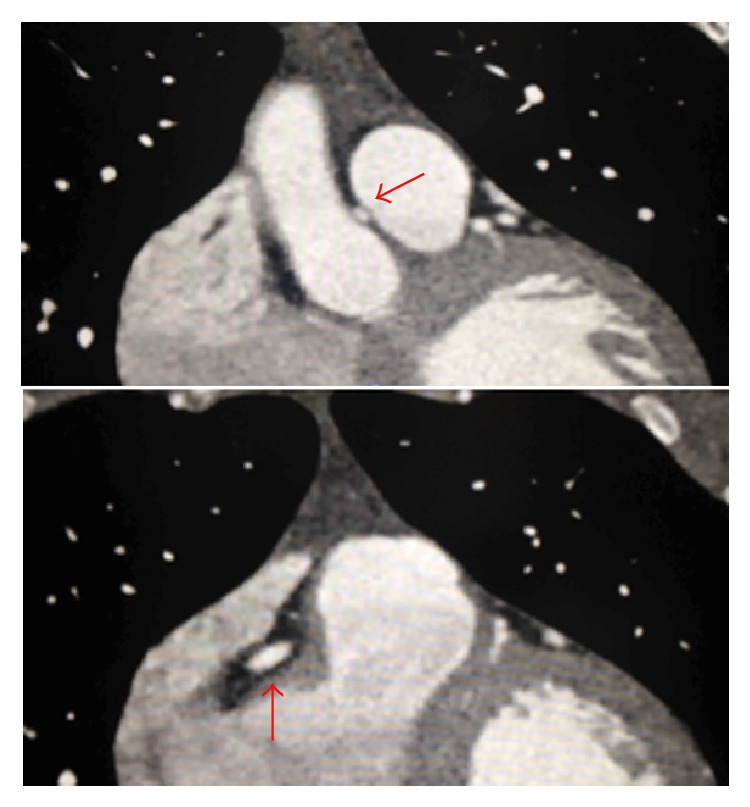
CCTA: the anomalous right coronary artery is taking an interarterial course (A). The anomalous coronary artery is coursing between right atrioventricular groove.

**Figure 3:**
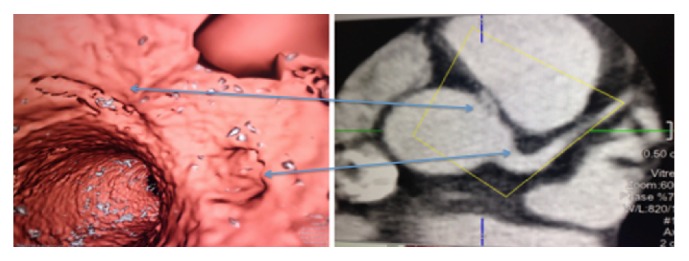
CCTA: 3D reconstruction of the interior slit-like opening and corresponding external 2D image.

**Figure 4:**
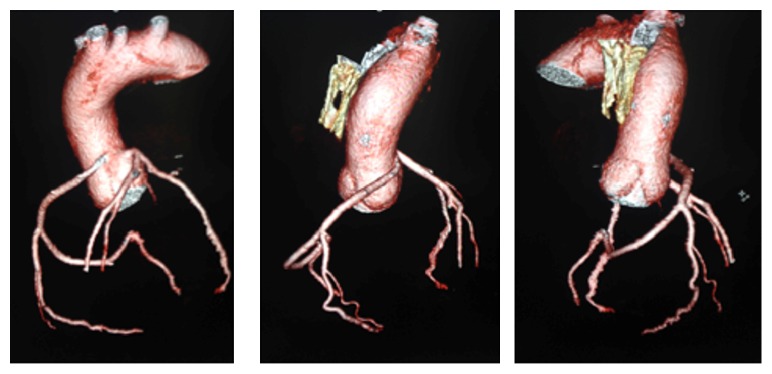
3D reconstruction of the vessels illustrating the course of the right anomalous coronary artery.

**Figure 5:**
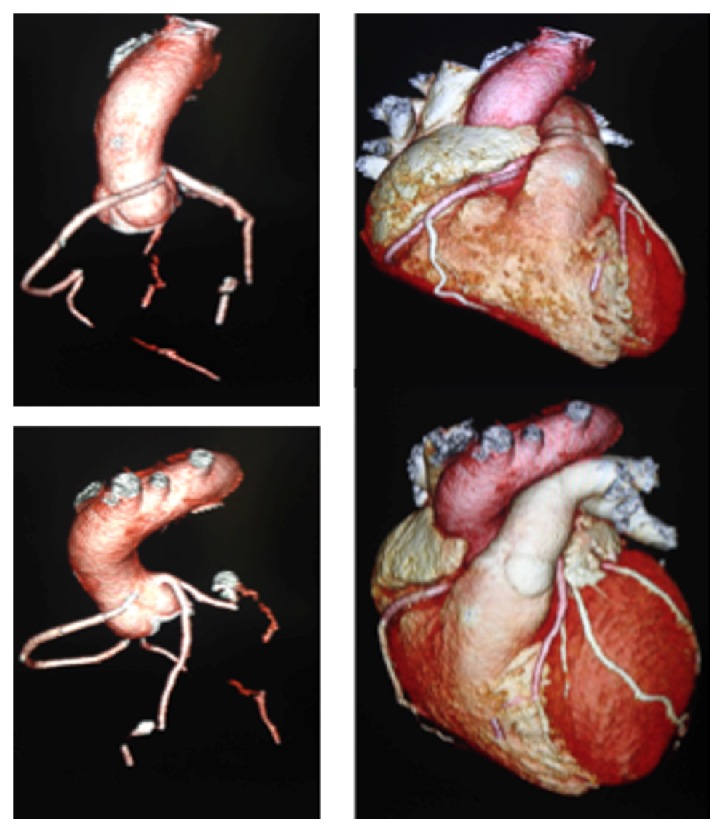
3D reconstruction of the coronary vessels and the corresponding images of the coronary vessels with the heart.

CCTA is a superior noninvasive imaging technique that can be used to diagnose and exclude coronary anomalies with high accuracy. In addition, it can provide precise information such as vessel course and orifice location [4]. CCTA images in Figures 1 and 2 are illustrating the course and anatomy of the right coronary artery in this patient.  Figure 3 is a 3D reconstruction of an internal view of the orifice of the right coronary artery highlighting the slit-like opening and intramural course of the right coronary in comparison to the circular orifice of the left coronary. Figures 4 and 5 are 3D reconstructions of the coronary arteries from various angles again depicting the course of the vessels and demonstrating the accuracy and details that can be obtained from CCTA images.

After the diagnosis is made, the next diagnostic approach is to evaluate for ischemia with a perfusion stress imaging such as either with a cardiac perfusion nuclear or MRI stress imaging. Nuclear stress testing is readily available in most centers; however, a cardiac MRI is a preferable first-choice modality if available as it is radiation-free and can assess for ischemic related subendocardial fibrosis, which is often missed by nuclear imaging.

One of the largest studies on ACAOS was conducted by American Armed Forces Institute of Pathology where 6.3 million 18-year-old recruits who underwent intense military training for 8 weeks were looked at for unrelated trauma deaths. There were 64 cardiac deaths and 21 (33%) were related to L-ACAOS. There was not any other coronary anomalies which lead to death (including R-ACAOS). This study showed that although the left anomalous coronaries are dangerous, the right coronary artery anomalies might not pose as big a risk of sudden death.

The conservative management options for ACAOS include observation, beta blocker medications, and avoiding strenuous activity. Invasive procedure for the treatment of ARCAOS includes stenting, CABG, unroofing, and reimplantation surgery. Stenting is associated with high risk of restenosis likely due to the phasic compression of the stent during normal cardiac cycle. CABG does not require opening the aortic root; however it is associated with poor long-term patency and presence of anterograde competitive flow. Unroofing surgery relieves systolic compression of proximal anomalous coronary artery and reimplantation creates neoostial orifice at the correct coronary sinus. There is still risk of stenosis of neoostial orifice and aortic valve injury. According to ACC/AHA 2008 recommendation for congenital coronary anomalies of ectopic arterial origin, the main indications for surgery in ACAOS include documentation of myocardial ischemia, vascular wall hypoplasia, and/or obstruction to coronary flow.

## 3. Conclusion

Many cases in literature describe right ACAOS in the setting where the patient experiences a major cardiac event or has clear coronary ischemic symptoms. The management course in these cases and in cases where myocardial ischemia is documented is clear-cut. However, in patients with vague nonlimiting symptoms, which could be cardiac in nature, and no clear evidence of myocardial ischemia, further investigation may be warranted.

Our patient underwent a submaximal dobutamine cardiac MRI stress test with gadolinium, which was negative for ischemia and infarction. Given that the maximum heart rate was not reached, the MRI stress test interpretations are limited. A year later, a Bruce protocol treadmill stress echocardiogram was performed and was also negative for myocardial ischemia. The patient in this case does not participate in competitive sports and opted for conservative management. The patient was advised to stop smoking and avoid strenuous exercise and placed on beta blockers for his hypertension. He will be followed up yearly for stress testing.
